# Allogeneic fresh frozen plasma eye drops for the treatment of ocular Graft-versus-Host disease: prospective open-label study

**DOI:** 10.1007/s00417-025-06870-1

**Published:** 2025-06-09

**Authors:** Yoav Nahum, Noa Golomb, Ronit Goldman-Levi, Michael Mimouni, Eitan Livny, Mali Rubinstein, Irit Bahar, Liat Shargian, Pia Raanani, Vered Yahalom, Moshe Yeshurun

**Affiliations:** 1 Department of Ophthalmology, Rabin Medical Center – Beilinson Hospital, 39 Jabotinsky St, Petach Tikva, 4941492 Israel; 2 Gray Faculty of Medical & Health Sciences, Tel Aviv University, Tel Aviv, Israel; 3 Blood Services & Apheresis, Rabin Medical Center – Beilinson Hospital, Petach Tikva, Israel; 4 Department of Ophthalmology, Rambam Health Care Campus, Haifa, Israel; 5 Bruce and Ruth Rappaport Faculty of Medicine, Technion-Israel Institute of Technology, Haifa, Israel; 6 Institute of Hematology, Davidoff Cancer Center, Beilinson Hospital, Rabin Medical Center, Petach Tikva, Israel

**Keywords:** Ocular surface, GVHD, Plasma

## Abstract

**Purpose:**

To evaluate the safety and efficacy of allogeneic fresh frozen plasma drops for the treatment of chronic ocular graft-versus-host disease (oGVHD).

**Methods:**

In this prospective open-label institutional study, adult oGVHD patients were administered allogeneic fresh frozen 100% plasma (FFP) eye drops QID or more for 3 months, in addition to the patients’ usual treatment. The drops were prepared using a closed sterile tubing system (COL© System, Biomed Device Srl., Italy). The main outcome measures included the Ocular Surface Disease Index (OSDI), corneal fluorescein staining National Eye Institute (NEI) scale, basal tear secretion test, and functional assessment of cancer therapy–bone marrow transplant (FACT-BMT) questionnaire.

**Results:**

This study included 25 patients (49 eyes). OSDI scores decreased from 53 ± 26 at baseline to 32 ± 22 after 1 month and 30 ± 23 after 3 months (*P* < 0.0001), and fluorescein staining NEI grade decreased from 7.04 ± 3.51 at baseline to 5.5 ± 3.1 after 1 month (*P* < 0.0001) and 5.3 + 3.1 after 3 months (baseline to 3 months, *P* < 0.0001; one month to 3 months, *P* = 0.64). Mean basal tear secretion was 4.65 ± 3.4 mm at baseline and 6.5 ± 4.7 mm after 3 months (*P* = 0.002). The FACT-BMT scores did not change significantly from baseline to 3 months (106.3 ± 22 vs. 112 ± 20, *P* = 0.08). A subset of eight patients who were using scleral or bandage contact lenses concurrently showed significant improvement, and four of them were managed without lenses during the study period. No significant adverse events were noted.

**Conclusion:**

Allogeneic FFP eye drops are safe and effective in the treatment of chronic oGVHD.

**Trial registration:**

The trial is listed from 10/2020 in the Israeli Ministry of Health Clinical Trials Registry (MOH_2021-09-30_010276).

## Key Messages

*****What is known***:**

- Several autologous and allogeneic blood products have been studied as a treatment for chronic oGVHD as well as for other ocular surface diseases. Allogeneic fresh frozen plasma was evaluated in-vitro but no clinical studies have examined its safety and efficacy for ocular surface diseases.

*****What is new***:**

- Our open label study showed that adding allogeneic FFP eye drops is safe and beneficial in the treatment of chronic oGVHD that were symptomatic despite standard treatment modalities.
- Significant improvement was also seen in the subset of patients who were concurrently using scleral or bandage contact lenses, and half of them were able to discontinue the use of lenses during the study.

## Introduction

Allogeneic hematopoietic cell transplantation (alloHSCT) is a curative therapy for a large number of malignant and nonmalignant hematological diseases. Graft-versus-host disease (GVHD) is a major cause of morbidity and mortality following alloHSCT. Acute GVHD occurs in up to 40% of patients undergoing alloHSCT, and chronic GVHD occurs in 30–70%, affecting the skin, mouth, liver, lungs, gastrointestinal tract, and eyes [1]. Ocular GVHD (oGVHD) has been reported in 40–90% of allogeneic bone marrow transplant recipients [2]. Ocular involvement ranges from mild dry eye syndrome, causing ocular irritation, to intense inflammation and desiccation, which can lead to sloughing of the corneal epithelium, stromal melting and perforation, total limbal stem cell deficiency, and scarring and keratinization of the ocular surface [2]. 

Blood-derived products, namely serum, platelet-rich plasma, plasma enriched with growth factors, platelet lysate, umbilical cord blood, and even whole blood, are time-honored treatments for the amelioration of signs and symptoms of oGVHD [3–5] as well as other forms of severe ocular surface disease. The therapeutic effects and their impact on the growth and proliferation of corneal epithelial cells are attributed to both their biochemical and physical properties, similar to those of natural tears (pH, viscosity, etc.), as well as their protein content, which includes epithelial growth factors such as epidermal growth factor (EGF), fibroblast growth factor, nerve growth factor, platelet-derived growth factor (PDGF), insulin-like growth factor, and anti-inflammatory mediators such as transforming growth factor-beta1 (TGF-β1) [3, 6]. However, autologous blood products have important limitations, including patient inconvenience and the presence of anti-self-immunity factors. Several studies have demonstrated the efficacy of allogeneic serum eye drops and umbilical cord blood for reducing the signs and symptoms of oGVHD [7–10]. 

Human fresh frozen plasma (FFP) is acellular fluid derived from whole blood or plasmapheresis. It contains all blood proteins, including clotting proteins such as fibrinogen. In contrast to serum, FFP is easily available from blood banks as a standardized product produced under standard operating procedures in a quality-controlled setup. However, several in vitro cell culture studies have demonstrated that FFP has a lower capacity than peripheral blood serum and cord blood serum to induce proliferation, differentiation, and migration of corneal epithelial cells [11–14]. The difference was attributed to the higher content of growth factors in serum (EGF, PDGF, TGF-β1, and fibronectin), which are released from activated platelets during the coagulation process. In contrast, plasma is obtained from anticoagulated whole blood or via plasmapheresis, in which intact platelets are removed from the liquid phase without the release of additional mediators. Other possible causes of the inferior epitheliotropic profile of FFP include lower levels of vitamins A and E compared to serum, and the antiproliferative effect of citrate, which is used as an anticoagulant in its production process [11–14]. To the best of our knowledge, FFP has not been clinically tested for the treatment of oGVHD or other ocular surface diseases. This study aimed to evaluate the effect of adding allogeneic preservative-free 100% FFP drops to adequately treat adults with oGVHD in addition to their usual treatment.

## Materials and methods

### Study design

This prospective open-label institutional study was conducted at the Rabin Medical Center (RMC), a tertiary university hospital in Israel, between May 2021 and August 2022. This study adhered to the tenets of the 1964 Declaration of Helsinki and was approved by the local ethics committee (approval no. RMC 0646 − 19). The trial is listed in the Israeli Ministry of Health Clinical Trials Registry (MOH_2021-09-30_010276(. Written informed consent was obtained from all patients prior to enrollment.

The study population was comprised of adult patients treated for oGVHD following allogeneic bone marrow transplantation at the RMC. All patients were symptomatic despite standard treatment provided by a cornea specialist, including systemic immunosuppression, lubrication agents and steroid drops, punctal plugs, or surgical occlusion. Some patients also received scleral or bandage contact lenses.

All patients were treated with 100% allogeneic FFP drops QID or more for three months. The drops were given in addition to their usual treatment; patients were permitted a change only in their lubricating agents or contact lenses. They were asked to record the use of FFP drops or any other ocular drops, gels, or ointments in a diary supplied at the study onset. Examinations were scheduled at entry to the trial and at one and three months after commencing treatment.

The main outcome measures were the results of a self-administrated Ocular Surface Disease Index (OSDI) questionnaire, which had undergone forward and back translation from English to Hebrew [15] and the corneal fluorescein staining National Eye Institute (NEI) scale [16]. The NEI scale was graded by two independent experienced cornea and external eye disease specialists. One grader (Y.N.) was unmasked and graded using a slit-lamp. Photographs of the stained corneas of each patient taken with the slit-lamp were then randomly shown to the second grader (M.M.), who was blinded to the time point at which the images were taken. The average of the two grades was considered as the final grade. In addition, the patients completed the Hebrew version of the Functional Assessment of Cancer Therapy-Bone Marrow Transplantation (FACT-BMT) quality-of-life questionnaire [17]. 

At patient visits, the following data were recorded: demographics and prior ophthalmic treatments (first visit), Snellen-corrected distance visual acuity, findings on basal tear secretion test and slit-lamp examination, intraocular pressure measurement, oGVHD National Institutes of Health (NIH) grade [18], OSDI, and FACT-BMT questionnaire scores.

## Preparation of allogeneic fresh frozen plasma eye drops

Plasma (200–600 ml) was collected from healthy donors at the RMC Blood Services and Apheresis Institute according to standard criteria using Trima Excel (Terumo^®^) or MCS+(Haemonetics^®^) apheresis instruments. Donor testing was performed according to the Israeli Ministry of Health guidelines. After the plasma was harvested, aliquots for use as eye drops were prepared using a closed, sterile COL© tubing system (Biomed Device Srl., Modena, Italy). A plasma bag containing 200 cc was connected to the COL device, and the plasma was divided into 50 1.45-ml bottles and frozen at −30^O^C in quarantine until donor testing was completed. For each batch, the sterility of the COL system was validated by Hy Laboratories (Yavne, Israel). If all donor results of transmittable disease tests (serology and nucleic acid testing) were non-reactive and no growth was detected in the sterility test, the FFP eye drops were packed in boxes, each containing 30 bottles and labeled using the same process as for other blood components. Thereafter, FFP drops were administered to patients. The blood group was matched to that of the patients prior to bone marrow transplantation. The drops were then placed in a cooler with ice packs. The patients were instructed to keep the eye drop bottles at −18^0^C in their home freezer, to use a new, thawed bottle every day, and to keep bottles in their refrigerator (4^0^C) while they were in use.

### Statistical analysis

All the data collected in the study were entered into an electronic database using Microsoft Excel 2007 (Microsoft Corp., Redmond, WA, USA). Patient characteristics are summarized as frequencies (number and percentages) for categorical variables and median (range) or mean (SD), as appropriate, for continuous variables. Snellen visual acuity values were converted to the logarithm of the minimum angle of resolution (logMAR) scale for statistical purposes. Data were analyzed using MedCalc Online Calculators (MedCalc Software, Mariakerke, Belgium). When appropriate, a paired Student’s t-test was used to analyze the quantitative measures. Differences were considered statistically significant when the *p* value was less than 0.05. Bonferroni correction for multiple comparisons was used as indicated. The sample size was based on an expected difference of seven points in OSDI scores from before to after treatment and on the standard deviations in the studies by Agomo et al. [15] and Suri et al. [19] comparing OSDI and NEI scores between patients with oGVHD and healthy subjects.

## Results

This study included 25 patients (49 eyes) with oGVHD after alloHSCT. The demographic details are presented in Table 1. Briefly, there were 14 male and 11 female patients with a median age of 59 years (range 25–77). The most common indication for alloHSCT was acute myeloid leukemia (*n* = 15), followed by acute lymphoid leukemia (*n* = 3), myelodysplastic syndrome (*n* = 2), and others (*n* = 5). Patients were enrolled in the study at a median of four years (range, 2–14) after transplantation. Upon enrollment, the GVHD NIH grade was mild in nine patients, moderate in 15, and severe in one. There were no changes in grade during the study period.

**Table 1:** Demographic and clinical characteristics of patients with oGVHD

No.	Sex	Age (yr)	Indication for BMT	Years since BMT	No. of plugged or cauterized puncta	Ocular medications other than lubrication*	Contact lens usage	NIH oGVHD grade
1	F	63.0	AML	3	2	FML twice a week		Moderate
2	F	44.0	ALL	9	3			Moderate
3	F	46.0	AML	14	4	Ofloxacin TID	Bandage	Mild
4	F	72.0	AML-MDS	8	2	Ofloxacin TID	Bandage	Moderate
5	M	59.0	AML	5	4	FML twice a week, Restasis BID		Moderate
6	F	52.0	AML	4	1	FML twice a week	Scleral	Moderate
7	M	56.0	MDS	3	4	Cyclosporine 0.05%	Scleral	Mild
8	M	77.0	AML	11	0			Mild
9	M	46.0	CLL DLBCL	3	4	Brimodine-timolol		Mild
10	F	42.0	MLBCL	6	0	FML once a day, Dorzolamide-timolol	Scleral	Mild
11	F	67.0	AML	11	0	Cyclosporine 0.05%		Severe
12	M	25.0	BALL	4	0			Moderate
13	F	67.0	PMF	4	4	FML twice a week		Moderate
14	M	67.0	MDS	3	0	FML twice a week		Moderate
15	M	74.0	AML	2	2	FML every other day		Mild
16	F	58.0	DLBCL	4	1	FML twice a week		Moderate
17	F	60.0	AML	7	4		Scleral	Moderate
18	M	69.0	AML	7	4	FML once a day		Moderate
19	F	67.0	AML	3	1	FML twice a week, Ofloxacin TID	Bandage	Moderate
20	M	47.0	AML	3	4	FML twice a week	Scleral	Mild
21	M	29.0	ALL	9	0	FML twice a week		Moderate
22	M	62.0	AML	2	2	FML twice a week		Moderate
23	M	30.0	AML	3	2	FML twice a week		Mild
24	M	53.0	CML	9	0	FML twice a week		Moderate
25	M	71.0	AML	7	2	Acetazolamide 250 mg once a day		Mild

*ALL *acute lymphocytic leukemia, *AML *acute myeloid leukemia, *BMT *bone marrow transplantation, *CLL *chronic lymphocytic leukemia, *CML *chronic myeloid leukemia, *DLBCL *diffuse large B cell lymphoma, *FML*^®^ fluorometholone eye drops, *FML *fluorometholone, *MDS *myelodysplastic syndrome, *MLBCL *mediastinal large B-cell lymphoma, *oGHVD *ocular graft-versus-host disease, *PMF *primary myelofibrosis, *BALL *B-cell acute lymphocytic leukemia, *FML *Fluorometholone

At baseline, all patients were administered a variety of lubricating agents. Fifteen patients were also treated with fluorometholone drops (FML Liquifilm 0.1%, Allergan PLC, Dublin Ireland), mostly once or twice daily during exacerbations, and then tapered to a maintenance dosage of twice a week. Three patients were using anti-glaucoma drops or oral anti-glaucoma medications, and 3, cyclosporine 0.05% drops BID. Three patients with bandage contact lenses (BCLs) also received ofloxacin drop TID. In 18 patients, one or more lacrimal puncta (all in eight patients) were plugged or cauterized. In addition to the 3 patients with BCLs, 5 patients were dependent on daily scleral contact lenses. None of the patients had used blood-derived eye drops in the year prior to the study.

**Fig. 1:**
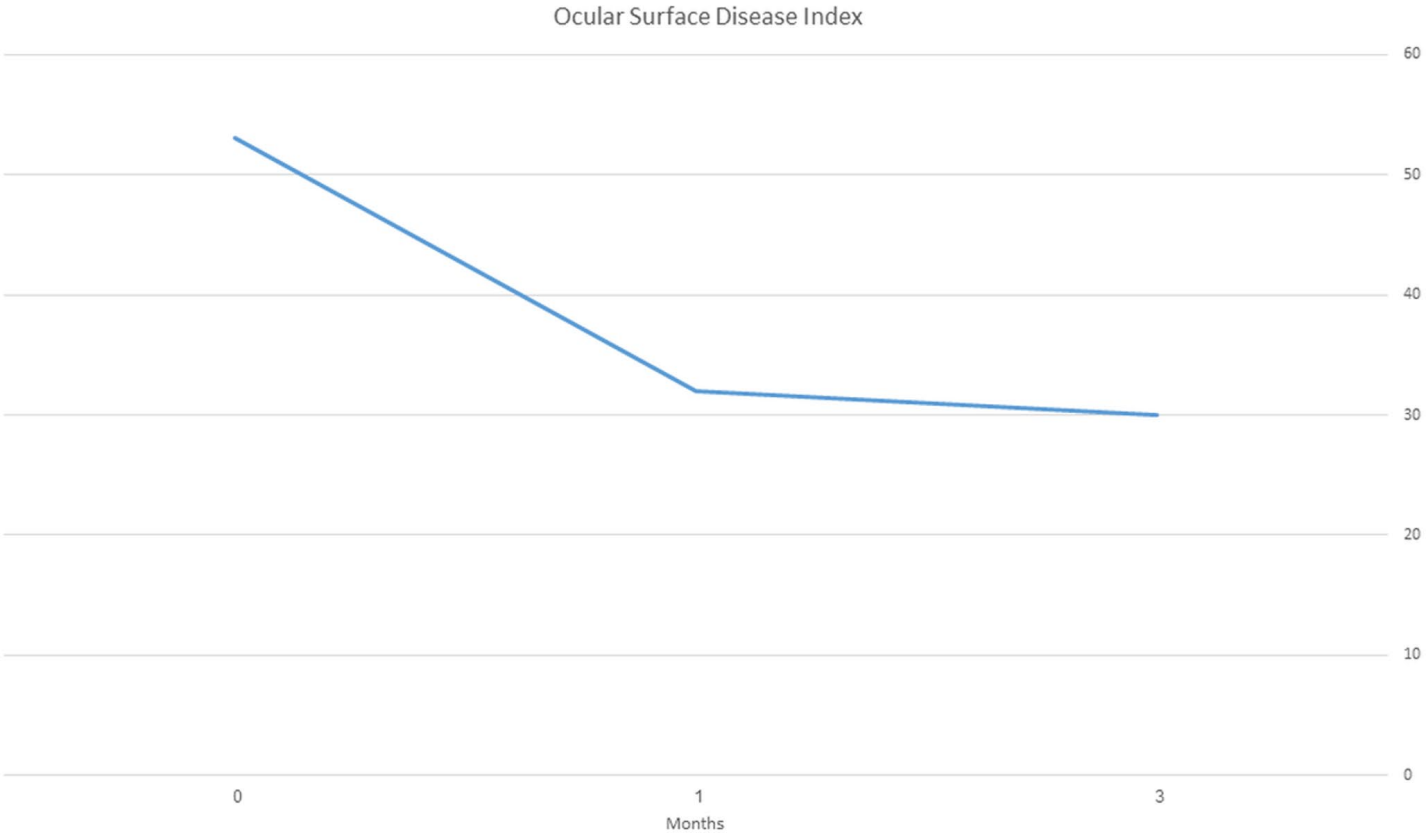
Ocular Surface Disease Index (OSDI) during plasma drops treatment. Change in Ocular Surface Disease Index scores during treatment with allogeneic plasma drops in oGVHD patients

Patients reported applying FFP drops 4–12 times daily (6.3 ± 2.3). Best-corrected visual acuity was logMAR 0.075 ± 0.096 at baseline and 0.06 ± 0.1 after 3 months (*P* = 0.42). Mean score on the basal tear secretion test increased significantly from 4.65 ± 3.4 mm at baseline to 6.5 ± 4.7 mm after 3 months (*P* = 0.002). The OSDI scores decreased significantly from 53 ± 26 at baseline to 32 ± 22 after 1 and 30 ± 23 after 3 months (*P* < 0.0001) (Fig. 1).

**Fig. 2:**
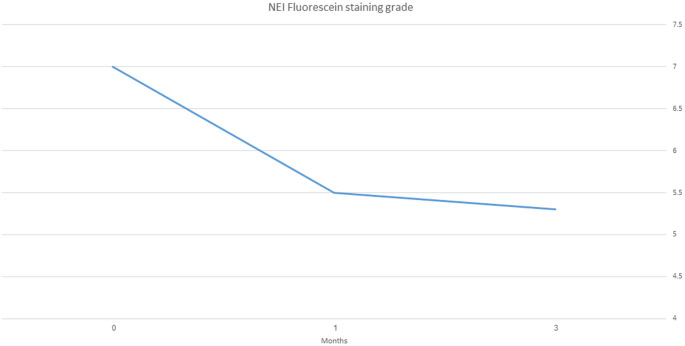
Corneal fluorescein staining during plasma drops treatment. Change in fluorescein staining National Eye Institute (NEI) scores during treatment with allogeneic plasma drops in oGVHD patients

In 49 eyes, fluorescein staining NEI grade decreased significantly from 7.04 ± 3.51 at baseline to 5.5 ± 3.1 after 1 month (*P* < 0.0001) and 5.3 + 3.1 after 3 months (baseline to 3 months, *P* < 0.0001; 1 month to 3 months, *P* = 0.64) (Fig. 2).

**Fig. 3:**
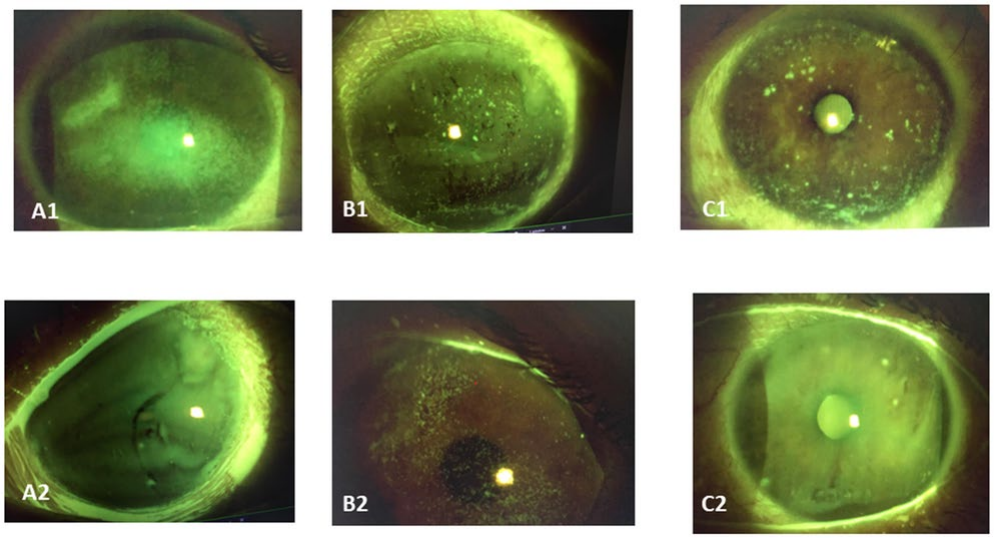
Fluorescein staining in three oGVHD patients before and after plasma drops. Fluorescein corneal staining of three eyes of oGVHD patients before (Figures A1,B1,C1) and 3 months following (Figures A2,B2,C2) allogeneic plasma drops treatment

When fluorescein staining grades were calculated for each grader separately, those of the second (masked) grader showed a significant decrease, from 6.17 ± 4.0 at baseline to 5.3 ± 3.1 after 1 month and 4.97 + 3.5 after 3 months of FFP use (*P* < 0.005). Of the 147 data points, 16 (10.9%) were not graded by the second grader because of insufficient image quality: one point at baseline, 10 points at the 1-month visit, and 5 points at the 3-month visit. Examples of fluorescein staining at baseline and after 3 months of treatment are shown in Fig. 3.

FACT-BMT scores showed an increase in borderline significance from baseline to 3 months (106.3 ± 22 vs. 112 ± 20, *P* = 0.08).

Significant improvement was also seen in the subset of 8 patients who were concurrently using scleral or bandage contact lenses. Four patients were managed without lenses during the study.

One patient experienced irritation after 2 months of treatment, which resolved a new batch of drops. No other adverse events were reported.

## Discussion

Treatment of chronic oGVHD is challenging for both patients and ophthalmologists. Our study population included patients with mild, moderate, or severe ocular surface disease who were being treated by a corneal specialist in a tertiary care center using all currently available treatment modalities that were shown to be effective in this disease such as steroids, cyclosporine and tacrolimus [20, 21]. Nevertheless, they were still symptomatic with constant ocular irritation that limited their activities and negatively affected their quality of life.

Following in vitro cell culture studies that found FFP to have a lesser epitheliotropic capacity than blood serum [11–14], FFP was, to the best of our knowledge, never evaluated clinically as a treatment for oGVHD or other ocular surface diseases, let al.one clinically compared to other blood products. Our results demonstrated that, although FFP does not contain growth factors released by platelets in comparison to serum, it is a potent therapeutic agent for oGVHD. We believe that this effect is largely attributable to its physical properties, such as pH, osmolarity, viscosity, and ability to adhere to the outer layer of the cornea, rather than to its protein content.

In addition to avoiding inconvenience to patients, there are several advantages of using FFP from healthy donors. The condition of patients varies with the course of the disease. They may be anemic, such that repeated blood withdrawal for tear production is not warranted, and their sera may contain different cytokines, extracellular vesicles, drugs, and self-antibodies [22]. At the same time, plasma donation by healthy donors is a regulated process performed worldwide using standard donor criteria, donation testing, production, quality control, storage, labelling, and component allocation procedures. Therefore, the production of allogeneic FFP drops can be integrated smoothly into this process. The drops can be manufactured using a 200 ml plasma bag, in a system separate from whole-blood donation. Plasma is obtained by apheresis concomitant with a plateletpheresis donation or using 600 ml plasma from a single donor, without the need to prepare serum and discard red blood cells.

A recent study reported that oGVHD is associated with a progressive decline in tear production even when other measures are improved [19]. However, in our patients, basal tear secretion increased significantly. Although it is unlikely that the treatment affected the main lacrimal gland located within the superotemporal aspect of the orbit, it might have affected the accessory lacrimal glands located in the conjunctival fornix and on the tarsal plate through its anti-inflammatory or growth factor activity.

In this study, FFP was ABO-matched to patients’ blood types prior to alloHSCT. Although therapeutic substances present in eye drops are known to undergo some systemic absorption, a previous small case series describing the use of non-ABO-matched allogeneic serum for patients with oGVHD did not report relevant adverse events such as hemolysis or systemic allergic reactions [8, 9]. Establishment of the safety of non-matched FFP in larger studies would further simplify the acquisition and allocation of FFP eye drops.

This study has several limitations. First, the single-arm, real-world design dictated that the patients were heterogeneous in terms of baseline treatment regimen, status of ocular surface disease, and lack of a control group. Second, our inclusion criteria did not stipulate that patients had a formal diagnosis of oGVHD by any one of several published grading systems, such as the International Chronic oGVHD (ICCGVHD) consensus group diagnostic criteria or the National Institutes of Health Consensus Criteria (NIH CC) [20]. Nevertheless, 23 of the 25 patients had baseline OSDI scores of 20 or more plus corneal staining and would therefore meet the Tear Film and Ocular Surface Society – Dry Eyes Workshop (TFOS DEWS) II definition, and the two outstanding patients were already being treated with punctal plugs, lubrication, and topical steroids [23]. Finally, eight patients used therapeutic, scleral, or therapeutic contact lenses on which they depended on daily activities. The lenses were removed a few minutes before corneal staining was measured, which may have affected the staining pattern observed by the graders. This was particularly relevant in four patients who had contact lenses at the baseline measurement but then managed without them once they started therapy with FFP drops. Finally, tear-breakup-time (TBUT) was not assessed in this study. Levy et al. reported in 2019 that treatment with autologous serum eye drops did not improve TBUT [24] and it could have been interesting to know whether allogeneic plasma based eye drops can have a different result.

In conclusion, this prospective, single-center study showed for the first time that allogeneic FFP drops are safe and effective for the treatment of oGVHD. We hope that further studies will prove FFP to be safe and effective for other ocular surface diseases and that the production of allogeneic FFP eye drops can be standardized and added to our treatment armamentarium.
